# Editorial: An update on pediatric skeletal system infections

**DOI:** 10.3389/fped.2023.1128126

**Published:** 2023-02-13

**Authors:** Pablo Yagupsky, Dimitri Ceroni

**Affiliations:** ^1^ Clinical Microbiology Laboratory, Ben-Gurion University of the Negev, Beer-Sheva, Israel; ^2^ Unité Orthopédie Pédiatrique et de Traumatologie Infantile, Service de Chirurgie Pédiatrique, Hôpitaux Universitaires de Genève, Genève, Switzerland

**Keywords:** osteoarthritis, children, epidemiology, etiology, diagnosis, therapy

**Editorial on the Research Topic** An Update on Pediatric Skeletal System Infections

Osteoarticular infections (OAIs) in pediatric patients are medical emergencies that require early diagnosis and adequate treatment to avoid severe septic complications, prolonged morbidity, and long-term functional disability (1). Although traumatic inoculation of bacteria into the joint space and bones or dissemination from a contiguous soft tissue focus may occur, in most cases, pathogens reach the skeletal tissues through the hematogenous route (2). Because the incidence of bacteremia is elevated in immunologically naïve young children, the incidence of septic arthritis and osteomyelitis is higher in early childhood (1).

Early diagnosis of skeletal infections, identification of their etiologic agent, and prompt administration of adequate antimicrobial therapy are cornerstones of managing the disease, avoiding clinical deterioration, and preventing permanent orthopedic sequelae (3). Traditionally, culture isolation of the etiologic agent, its identification, and determination of its antibiotic susceptibility was performed by conventional methods. However, causative organisms may be present in the specimen at low concentrations, difficult to isolate, and/or children may have been empirically administered antibiotics before cultures were obtained. Consequently, many pediatric joint and bone infections remained bacteriologically unconfirmed (4). Because the microbiology laboratory results are obtained with a 2–3-day delay and the danger of administering an inappropriate or poorly efficient antibiotic, the initial therapy of bone and joint infections usually consists of wide-spectrum antimicrobial drugs, including those that provide appropriate coverage of *Staphylococcus aureus* (3). To ensure therapeutic success, antibiotics are administered through the intravenous route in the early phase and switched to oral therapy after body temperature has normalized for 24 h, local findings and motion have improved, and C-reactive protein (CRP) levels have decreased (1).

Technological progress has revolutionized the epidemiology, diagnosis, and management of pediatric OAI in recent decades, as summarized in Table 1.

**Table 1:** Main developments that have changed the management of pediatric skeletal system infections in recent years.

Area	Game-changing development	References
Epidemiology and etiology	Vaccination against *Haemophilus influenzae* type b and *Streptococcus pneumoniae*	(5, 6)
	Emergence of Panton-Valentine leukocidin (PVL)-producing community-associated methicillin resistant *S. aureus* (MRSA)	(7)
	Recognition of *Kingella kingae* as the prime etiology of skeletal system infections in the 6–48-months old group	(8)
	Realization that the etiology of pediatric osteoarthritis is age-dependent	(8)
Clinical features	Recognition of the mild local and systemic inflammation characteristics of *K. kingae*'s OAIs	(9, 10)
	Use of serum CRP levels to assess clinical response and shift from parenteral to oral antibiotics	(1, 3)
Diagnosis	Use of MRI to improve diagnosis of bone infections and determine their extent	(11)
	Development of improved culture methods and nucleic acid amplification tests	(12–14)
	Rapid identification of the isolate by molecular methods and MALDI-TOF technology	(15)
	Emergence of the metagenomic next-generation sequencing as a microbiological diagnostic tool	(16)
Therapy	Increasing use of oral therapy, replacing the traditional prolonged intravenous antibiotic regimens	(17, 18)
	Abolishing the need to determine serum cidal levels to shift from parenteral to oral therapy	(1, 19)

The present Special Issue provides an update on managing these skeletal system infections in children and the areas of controversy and current research to improve the diagnosis, shorten the duration of intravenous therapy, and reduce hospitalization length.

The article by Porsch et al. summarizes our current knowledge of the virulence factors of *Kingella kingae*. Advances in the culture methods and especially the increasing use of molecular detection methods have resulted in recognition of the bacterium as the prime etiology of hematogenous septic arthritis, osteomyelitis, spondylodiscitis, and tenosynovitis in children aged 6–48 months (8). Intensive research conducted over the last two decades has identified a wide range of bacterial surface factors that play crucial roles in the transition from the colonized oropharyngeal surfaces to the invasion of the skeletal system tissues (20).

The review by Searns et al. covers the highly debated issue of delaying the administration of antimicrobial therapy to children with suspected joint and bone infections. Postponing the initiation of antibiotics enables to obtain of surgical specimens for the identification of the pathogen and determining its antibiotic susceptibility. However, this delay may result in the patient's clinical deterioration. The increasing prevalence of the highly virulent and multiresistant PVL-positive MRSA in many regions requires a clear-cut answer (7). Septic children are at high risk for life-threatening complications and mortality and require immediate initiation of intravenous antibiotics. For well-appearing children, additional evidence is needed to define what period is acceptable to withhold antimicrobial therapy while awaiting surgical, diagnostic procedures.

Alcobendas et al. legitimately question the traditional recommendations of OAI treatment and suggest treating the patient individually, advocating short courses or no intravenous therapy (21). They highlight growing evidence of good outcomes in patients with primary OAI treated with a minimally invasive approach consisting of stricter surgical indications and short courses or no intravenous therapy. They consider that most children with *K. kingae* OAI do not usually require invasive surgical procedures to achieve clinical improvement. In contrast, invasive surgery remains indicated when causal pathogens are pyogenic and express specific virulence genes. The same concept is thereafter applied to the antibiotic treatment; if some children with severe OAI are more likely to respond better in children who initially received intravenous antibiotics, an exclusively oral administration could be a safe option in patients with OAI caused by *K. kingae*.

De Marco et al. articulate a full reflection about a better understanding of the etiology of OAIs, which could lead to major changes in their therapeutic management. They underline that it currently needs to be a consensus about the treatment of OAIs, especially about who can be safely treated solely using medicines and who requires and will benefit from a surgical approach. The indications that justify, in their opinion, the performance of a surgical procedure are reviewed. In the authors’ opinion, there are three basic indications for surgical procedures during pediatric OAIs: obtain a bacteriological diagnosis, ensure control of the infectious source, and preserve the maximal function of the affected bone segment. They advise therapeutic management according to the causal germs, and surgery remains essential when pyogenic bacteria are incriminated.

Finally, Moez et al. propose a new strategy for investigating spondylodiscitis in children younger than 4 years. The new diagnostic approach is based on the premise that detecting *K. kingae* RTX toxin genes in the oropharynx by swab real-time PCR assay provides strong evidence that this microorganism is responsible for the patient's OAI (22). This study demonstrated that *K. kingae*'s RTX toxin operon is detected on the oropharynx of approximately 90% of toddlers with confirmed spondylodiscitis. These results provided robust arguments that *K. kingae* should be considered as the primary etiology of spinal infections in children aged 6–48 months. The oropharyngeal swab PCR assay could become an early decision-making tool for indirectly identifying the etiology of spondylodiscitis where needle aspiration or open biopsy cannot be regarded as desirable diagnostic procedures due to their poor performance and surgical/anesthetic risks.

We want to thank all the authors and reviewers for their valuable time and contributions that made this publication possible.
