# Supplementary material for: Editorial: An update on pediatric skeletal system infections
Source: Front Pediatr. 2023 Feb 13;11:1128126. doi: 10.3389/fped.2023.1128126 (PMC9969156; doi:10.3389/fped.2023.1128126)
Supplement: Supplementary file 1 [file Table1.docx]

| **Area** | **Game-Changing Development** | **References** |
| --- | --- | --- |
| Epidemiology and Etiology | Vaccination against *Haemophilus influenzae* type b and *Streptococcus pneumoniae* | 5, 6 |
|  | Emergence of Panton-Valentine leukocidin (PVL)-producing community-associated methicillin resistant *S. aureus* (MRSA) | 7 |
|  | Recognition of *Kingella kingae* as the prime etiology of skeletal system infections in the 6-48-months old group | 8 |
|  | Realization that the etiology of pediatric osteoarthritis is age-dependent | 8 |
| Clinical Features | Recognition of the mild local and systemic inflammation characteristics of *K. kingae*’s OAIs | 9, 10 |
|  | Use of serum CRP levels to assess clinical response and shift from parenteral to oral antibiotics | 1, 3 |
| Diagnosis | Use of MRI to improve diagnosis of bone infections and determine their extent | 11 |
|  | Development of improved culture methods and nucleic acid amplification tests | 12-14 |
|  | Rapid identification of the isolate by molecular methods and MALDI-TOF technology | 15 |
|  | Emergence of the metagenomic next-generation sequencing as a microbiological diagnostic tool | 16 |
| Therapy | Increasing use of oral therapy, replacing the traditional prolonged intravenous antibiotic regimens | 17, 18 |
|  | Abolishing the need to determine serum cidal levels to shift from parenteral to oral therapy | 1, 19 |

**Table 1.** Main developments that have changed the management of pediatric skeletal system infections in recent years
